# Supplementary material for: Physiologically relevant real-world light exposure and its behavioural and environmental determinants in Kumasi, Ghana
Source: Open Res Eur. 2025 Sep 26;5:300. [Version 1] doi: 10.12688/openreseurope.21304.1 (PMC12521892; doi:10.12688/openreseurope.21304.1)
Supplement: Peer review history

# Reviewer report: Reviewer response for version 1

**DOI:** 10.21956/openreseurope.23041.r61414

**Published:** 2025-10-22

**Authors:** Myriam Aries

**Affiliations:** 1 Jönköping University, Jönköping, Sweden

The paper describes the gathering of a unique dataset collected in Kumasi, Ghana, between October 2024 and February 2025, featuring personal light exposure measured with wearable melanopic light loggers, alongside behavioural and environmental data from self-reports. Fifteen participants wore three devices continuously for seven days (near-corneal plane, chest, and wrist).

 Participants completed multiple daily and start/end-of-study questionnaires and structured assessments of their sleep environment and light-related behaviour. The dataset, already available online, is organized into two main categories: group-level and individual-level data and will later be augmented into a standardized format.

 The paper presents the applied methodology in a thorough and well-documented manner, complemented by the open availability of the dataset. This reflects a clear and commendable commitment to transparency and reproducibility.

 The following suggestions might enhance overall motivation and reporting of the applied methodology and data set.

 The **Title** may be somewhat misleading, as the paper primarily presents the study protocol and applied methodology without reporting or analyzing results (there is referral to online datasets). It would be beneficial if the title more accurately reflected this focus.

 In the **Abstract**, the final sentences suggest that the applied methodology and collected dataset could “enable cross-context comparisons”, which is indeed a valuable potential. However, this implication may be slightly misleading, as such comparisons are not demonstrated within the current manuscript.

 In the **Introduction**, it would be valuable to emphasize why measuring light exposure in a country like Ghana is particularly interesting. Located near the equator (Accra 05°33″N 00°11′33″W), Ghana experiences a tropical savanna climate with consistently high temperatures and two rainy seasons, creating comfortable outdoor conditions for much of the year. This climate supports a traditionally outdoor-oriented lifestyle, where daily activities such as markets, food preparation, and social gatherings often occur outside, offering increased opportunities for daylight exposure. Additionally, Ghana’s near-equatorial position results in relatively stable photoperiods of approximately 12 hours throughout the year, which may have implications for circadian entrainment and light-based interventions.

 In the **Method** part, highlight why the triple measurement position is chosen and motivate why it can be of interest to have all three input values, and not only the one at eye level.

 The geographic coordinates for the field study location Kumasi may have large accuracy and, for example, 6°68´ N, -1°57´ W (degrees and minutes) will be sufficient to report, in particular since participants were allowed to come from (and likely move around) 60 km of the research centre.

 The study asked, via the Assessment of Sleep Environment (ASE) questionnaire, about factors influencing the sleep quality and the sleep environment. How were potential other influencing factors (i.e., a party, sickness of a family member) reported?  A party can mean longer than usual outdoor exposure, and a sick family member can mean longer than normal indoor exposure.

Are sufficient details of methods and materials provided to allow replication by others?

Yes

Is the rationale for creating the dataset(s) clearly described?

Yes

Are the datasets clearly presented in a useable and accessible format?

Yes

Are the protocols appropriate and is the work technically sound?

Yes

Reviewer Expertise:

Lighting Science and Technology, Building Physics and Environmental Design, Human Factors in Lighting

I confirm that I have read this submission and believe that I have an appropriate level of expertise to confirm that it is of an acceptable scientific standard.

# Reviewer report: Reviewer response for version 1

**DOI:** 10.21956/openreseurope.23041.r61406

**Published:** 2025-10-14

**Authors:** Angus Burns

**Affiliations:** 1 Brigham and Women’s Hospital, Boston, MA, USA

This work presents a well-designed and timely open dataset characterizing real-world light exposure patterns among young adults in Kumasi, Ghana. This work proposes to be the first account of its kind, measuring light exposure in free-living human beings under naturalistic conditions from sub-Saharan Africa. Using three ActLumus light loggers placed near the corneal plane, chest, and wrist, participants’ light exposure was recorded (seven days continuous). Participants provided self-reported measures of sleep, mood, exercise, and light-related behaviors. By integrating objective, high-frequency light data with ecological momentary assessments and validated questionnaires (e.g., MEQ, MCTQ, WHO-5), the dataset enables sophisticated analyses of light, mood and behavior relationships under naturalistic conditions. Small methodological reporting issues are raised below. Further expansion of this dataset to a more demographically diverse population and simply a larger sample will dramatically improve its value. 

 The study’s strengths lie in the novelty of its sample, rigorous methodology for light measurement and commitment to FAIR (Findable, Accessible, Interoperable, Reusable) data principles. The open-source repository structure fosters reproducibility and encourage reuse for cross-country comparisons through the broader MeLiDos network. 

 Nonetheless, the dataset is quite small and demographically narrow ( *n *= 15, mean age ≈ 22 years) which limits generalizability somewhat but this is an excellent start.

 Minor methodological clarifications should be updated for the revision:

 1. Wear/non-wear flagging methodology should be included and how this was handled in the resultant data.

 2. Replication scripts for summary plots 

 3. Why not use the actiwatches for sleep timing/duration measurement/reporting and include in the dataset? Contemporaneous measurement can be very useful for analysis.

 4. For the claim of this being the first work on this topic, perhaps include a database search reference (date; terms)

 Overall, this work is a valuable contribution to global circadian and environmental health research. It bridges a major geographic gap in personal light exposure data and provides a robust framework for future comparative and mechanistic studies.

Are sufficient details of methods and materials provided to allow replication by others?

Yes

Is the rationale for creating the dataset(s) clearly described?

Yes

Are the datasets clearly presented in a useable and accessible format?

Yes

Are the protocols appropriate and is the work technically sound?

Yes

Reviewer Expertise:

Sleep, circadian rhythms, genetics, actigraphy, epidemiology, statistics

I confirm that I have read this submission and believe that I have an appropriate level of expertise to confirm that it is of an acceptable scientific standard.

# Reviewer report: Reviewer response for version 1

**DOI:** 10.21956/openreseurope.23041.r61413

**Published:** 2025-10-14

**Authors:** Julia Ribeiro da Silva Vallim

**Affiliations:** 1 Universidade Federal de São Paulo, São Paulo, Brazil

**Summary of the Article**

 The data note under review is a valuable and novel investigation of real-life light exposure patterns in a specific demographic and geographic context: residents of Kumasi, Ghana. The authors supplement objective light logger data with subjective measures of sleep, light perception, and mood, resulting in a comprehensive and distinctive dataset. The thorough dataset description includes group- and individual-level data that are well-presented and informative. The study's novelty and its potential to serve as a reference for future work in this field are clear strengths.

**Overall Evaluation**

 The authors successfully addressed most of my concerns as a reviewer. The procedures and data presentation are sound and meaningfully contribute to the scientific literature. One key methodological aspect, however, requires clarification to ensure the article's scientific rigor and reproducibility.

 1. Clarification of "Normal Vision" Eligibility Criteria

 The authors state that participants were required to have "normal vision," but they do not define this term. Was it based on a previous diagnosis? Or was it based on self-reporting of no ocular diseases? Do the criteria for "normal" vision differ between younger and older adults?

Are sufficient details of methods and materials provided to allow replication by others?

Partly

Is the rationale for creating the dataset(s) clearly described?

Yes

Are the datasets clearly presented in a useable and accessible format?

Yes

Are the protocols appropriate and is the work technically sound?

Yes

Reviewer Expertise:

Sleep and Circadian Medicine

I confirm that I have read this submission and believe that I have an appropriate level of expertise to confirm that it is of an acceptable scientific standard.
